# Integrated Transcriptome and Metabolome Analysis of Fruit Quality Variation in ‘Sweet100’ Tomato Across Different Growth Stages

**DOI:** 10.3390/foods15050883

**Published:** 2026-03-04

**Authors:** Chunxin Liu, Congmin Wang, Shuya Xie, Yue Wang, He Zhang, Dalong Li, Tingting Zhao, Xiangyang Xu

**Affiliations:** 1 College of Horticulture and Landscape Architecture, Northeast Agricultural University, Harbin 150030, China; llucky992025@163.com (C.L.); 15954153922@163.com (C.W.); 19861241252@163.com (S.X.); yuewang20241015@163.com (Y.W.); esculentum@neau.edu.cn (H.Z.); lidalong@neau.edu.cn (D.L.); 2 Key Laboratory of Biology and Genetic Improvement of Horticultural Crops (Northeast Region), Ministry of Agriculture and Rural Affairs, Northeast Agricultural University, Harbin 150030, China

**Keywords:** tomato, fruit spike, growth stages, fruit quality

## Abstract

Previously, research has primarily focused on how the environment affects fruit quality, but there is a lack of studies investigating the impact of different growth stages on fruit quality. In this study, a total of 192 differentially abundant metabolites and 5546 differentially expressed genes were categorized into eight modules exhibiting distinct trends, along with an additional module that remained unchanged throughout all growth stages. These modules elucidate the primary metabolic alterations and transcriptional regulatory networks underlying quality variations in tomato fruits at the mature stage across different growth stages (Spike1–Spike3). Furthermore, an investigation was conducted on the module that remained constant throughout the growth stages. It was observed that the soluble sugar content remained stable across the different growth stages, whereas the levels of total phenols and flavonoids exhibited significant variation. Additionally, the principal metabolites influencing tomato flavor—namely aspartic acid, glutamic acid, glucose, fructose, citric acid, α-linolenic acid, and linoleic acid—did not demonstrate significant changes in content. The findings of this study provide novel insights into the formation of tomato quality and establish a theoretical foundation for the cultivation of long-season tomatoes with stable fruit quality.

## 1. Introduction

The tomato (*Solanum lycopersicum*) is recognized as the most economically significant fruit on a global scale [1]. Its fruits are abundant in titratable acids, phenolic compounds, γ-aminobutyric acid (GABA), and flavonoids, which confer anti-inflammatory, antioxidant, and anticancer properties [2,3,4]. Recent advancements in agricultural technology have enhanced tomato cultivation and management practices, leading to the development of numerous varieties suitable for extended growing seasons. As a result, the harvest period has been prolonged, with modern cultivation techniques yielding 5 to 6 clusters per plant, compared to the previous 3 to 4, and even more in some advanced agricultural settings [5,6]. In light of the increasing production of tomatoes, maintaining consistent fruit quality across various growth stages has emerged as a critical challenge for food quality control and sustainable horticultural production. This is particularly important given the role of tomatoes as a staple fresh food and a primary raw material in the global food supply chain. Although previous research has predominantly examined the environmental impacts on fruit quality, there is a notable deficiency in systematic studies exploring the influence of different growth stages on the nutritional and flavor quality of tomatoes.

Recent research has revealed notable variations in the volatile components and their concentrations at different locations within a single fruit spike, as well as among various anatomical parts of the tomato fruit. Specifically, there is substantial variation in total sugar and phenolic content across different fruit positions on the same spike, with the pericarp exhibiting a higher volatile content compared to the placental gel-like substance [7,8]. Furthermore, the concentration of hexose is greater in the tomato placenta gel than in the pericarp tissue [9]. The asynchronous growth and development of fruits across different spike layers lead to differences in water and nutrient transport provided by the plant to the fruit in each layer [10]. However, these studies did not elucidate the changes in metabolites across different growth stages, nor did they investigate the metabolite module that remains consistent throughout the entire growth period.

The Series Test of Cluster (STC) is an effective tool for identifying biomolecular modules with coordinated change trends and is extensively utilized in research fields such as plant development regulation, quality formation, and stress response. In studies investigating the healing mechanisms of plant grafting, STC was employed to conduct a dynamic transcriptome analysis of the callus formation process, elucidating the core regulatory function of photoreceptor A signal transduction factor 1 (PAT1) in cell proliferation and differentiation at the graft interface [11]. In research focused on the development of kiwifruit (*Actinidia chinensis Planch*) fruits, transcriptome data were categorized into 12 expression clusters using the STC method. This facilitated the construction of a spatio-temporal metabolic regulatory network for kiwifruit fruit development, clarifying the core regulatory modules at various developmental stages [12]. Similarly, during the ripening process of tomato fruits, STC was applied to organize transcriptome and metabolome data, successfully identifying co-expressed modules associated with the synthesis of key volatile flavor compounds, such as esters and terpenes [13]. In examining the mechanism of color formation during the maturation stage of wheat (*Triticum aestivum*) grains, a dynamic pattern analysis of differentially expressed genes using STC revealed that grain color differentiation is predominantly influenced by the temporal expression of anthocyanin synthesis pathways and associated regulatory genes [14]. The STC analysis further facilitated the investigation of metabolic and transcriptomic alterations induced by drought stress, elucidating the mechanisms of specialized metabolite changes in *Sophora alopecuroides* under mild, moderate, and severe drought conditions [15]. Despite its extensive application in research on plant quality formation and development, STC has not yet been employed to systematically study the accumulation patterns of metabolites and genes across various tomato growth stages. Addressing this research gap is essential for understanding the mechanisms underlying the stability of tomato fruit quality.

To address the identified research gaps and respond to the pressing need for consistent fruit quality in long-season tomato cultivation, this study undertook a comprehensive analysis of transcriptomic and metabolomic data derived from tomato fruits at the mature stage, specifically from growth stages Spike1 to Spike3. These stages correspond to distinct phases of tomato plant development. Eight metabolic and gene modules were constructed. By examining the phenylpropanoid and flavonoid biosynthesis pathways, the study elucidated the accumulation patterns of metabolites and gene expression across different growth stages. The findings offer precise molecular targets for breeding tomato varieties with enhanced uniformity in fruit quality and advance the understanding of the regulatory mechanisms governing metabolic accumulation across developmental stages. This research thereby provides theoretical support for the sustainable production of high-quality, long-season tomatoes.

## 2. Materials and Methods

### 2.1. Plant Materials and Sample Collection

‘Sweet100’ is a high-quality F1 hybrid tomato, which is the common “strawberry tomato” in the market. Tomato plants were cultivated within a standard greenhouse environment under meticulously controlled conditions, including a temperature regimen of 25 °C during the day and 18 °C at night, a relative humidity range of 60% to 70%, and a photosynthetic photon flux density of 300 μmol·m^−2^·s^−1^. The cultivation medium comprised a peat-perlite substrate in a 2:1 volume ratio. An integrated irrigation and fertilization system was employed, delivering a balanced nutrient solution with an N-P-K ratio of 15-5-30 every three days. The greenhouse was equipped with an automatic ventilation system that activated when temperatures surpassed 28 °C. All plants were uniformly managed under an identical cultivation protocol. To ensure uniform fruit development and mitigate the impact of fruit load, each plant was maintained with an identical number of fruits. The spatial arrangement of the plants within the greenhouse was completely randomized. Notably, the different fruit spikes (Spike1 to Spike5) of tomato plants correspond to different growth stages of the tomato during the long-season cultivation period. Tomato fruits were harvested 46 days post-anthesis, aligning with the fully ripe stage as established in prior research [16]. For each spike position (Spike1–Spike3), five uniform plants were selected, and fruits from the same spike were pooled and homogenized to reduce individual variation. Three independent biological replicates were prepared for each group, with all samples subsequently frozen in liquid nitrogen and stored at −80 °C for subsequent transcriptomic and metabolomic analyses.

### 2.2. Quality-Related Metabolic Analysis

#### 2.2.1. Total Soluble Solids and Titratable Acidity Analysis

A handheld digital refractometer (model PR-101, ATAGO, Tokyo, Japan) was used to measure the total soluble solids and titratable acidity of the mashed fruit [16].

#### 2.2.2. GABA, Total Flavonoids, Total Phenols and Lycopene Analysis

The quantification of gamma-aminobutyric acid (GABA) was achieved through a colorimetric assay involving the use of Streptomyces-derived GABA aminotransferase (GABA-AT), glutamate oxidase, and peroxidase [17]. This method utilized GABA-AT from *Streptomyces decoyicus* NBRC 13,977 in conjunction with glutamate oxidase and peroxidase. A 50 μL sample underwent pretreatment with 0.5 mL of Solution I at 30 °C for 10 min to eliminate potential interferences, followed by a reaction with 0.5 mL of Solution II at 30 °C for 20 min. The absorbance of the resulting chromogenic product was measured at 555 nm. The total flavonoid content was assessed using diethylene glycol analysis [18]. 20 μL extract was reacted with 4 M NaOH and 90% diethylene glycol at room temperature for 10 min, and the absorbance was recorded at 420 nm. The total phenolic content was determined via the Folin–Ciocalteu method [18]. 100 μL extract was mixed with 10% Folin–Ciocalteu reagent and sodium carbonate, reacted at room temperature for 10 min, centrifuged (3000× *g*, 5 min), and the supernatant absorbance was measured at 765 nm. The lycopene contents were carried out by the modified method used by previously described [19]. Lycopene was extracted from 0.5 g tomato sample with n-hexane (dark, 24 h, 25 °C), and the extract absorbance was measured at 503 nm for quantification against a lycopene standard curve. To ensure adequate statistical power and reliability, 15 independent biological replicates were used for each group in this study. This sample size is sufficient to achieve a statistical power ≥ 85% at a significance level of α = 0.05. All samples were randomly collected and uniformly handled to minimize systematic error.

#### 2.2.3. Fruit Firmness and Fruit Weight Analysis

The weight of each tomato fruit was measured using a scale with a precision of one-hundredth of a gram (SOPTOP, Shanghai, China) [20]. The firmness of the fruits was assessed utilizing a fruit hardness tester (Bareiss, Berlin, Germany) [21].

### 2.3. Transcriptome Data Analysis

The resulting cDNA library was sequenced using Illumina Novaseq6000 by Gene Denovo Biotechnology Co. (Guangzhou, China). Quality control (QC) was performed to filter low-quality reads and adaptor sequences. HISAT2 (v2.2.1) was used to map clean reads to the tomato ITAG4.0 reference genome. The Transcripts Per Kilobase of Exon Model per Million Mapped Reads (TPM) value was calculated for each transcription region using RSEM software and default parameters. Genes or transcripts with a false discovery rate (FDR) below 0.05 and an absolute fold change of at least 2 were classified as differentially expressed genes (DEGs) [22,23]. Multiple testing was adjusted using the Benjamini–Hochberg method in addition to FDR correction.

### 2.4. Metabolome Data Analysis

Individual tomato fruits (100 mg) were ground with liquid nitrogen, and the homogenate was resuspended in prechilled 80% methanol and 0.1% formic acid. The supernatant was injected into the LC-MS/MS system for analysis [24]. LC-MS/MS analyses were performed using an ExionLC™ AD system (SCIEX) coupled with a QTRAP^®^ 6500+ mass spectrometer (SCIEX) in Genedenovo (Guangzhou, China). Metabolites were identified by searching internal database and public databases (MassBank, KNApSAcK, HMDB, MoTo DB, and METLIN) and comparing the m/z values, the RT, and the fragmentation patterns with the standards. Hierarchical cluster analysis was performed, and the Pearson correlation coefficient was calculated to identify the differentially accumulated metabolites (DAMs) between the groups based on variable importance in projection (VIP) ≥ 1 and an absolute Log2FC (fold change) ≥ 1.

### 2.5. Principal Component Analysis (PCA) and Cluster Analysis

PCA plots and Hierarchical clustering were performed using the Metware Cloud, a free online platform for data analysis (https://cloud.metware.cn).

### 2.6. Transcriptome and Metabolome STC Analysis and Module Correlation Analysis

In order to detect the Expression pattern of DEGs or metabolites, the expression data of each sample (in processing order) were normalized to 0, log2 (v1/v0), log2 (v2/v2/v0), and then clustering was performed by ShortTime-series Expression Miner. For STC analysis, Spike1 was used as the reference condition to calculate expression ratios. Batch effects were corrected during data normalization. KEGG functional enrichment analysis was performed for metabolites and DEGs in each trend, and *p* values were calculated by hypothesis testing. After the obtained *p* value is corrected by FDR, KEGG that meets the threshold of Q value ≤ 0.05 is defined as KEGG that is significantly enriched in this trend [25]. Genes and metabolites were matched based on their shared KEGG pathway memberships and direct metabolic regulatory relationships. Pearson correlation coefficients (PCC) between module eigengenes were calculated, with PCC > 0.8 and *p* < 0.05 as the threshold for significant correlations. Significant module pairs were visualized in a polar coordinate plot, and validated by consistent expression trends and significant KEGG enrichment (Q ≤ 0.05).

### 2.7. Statistics

All experiments were independently replicated at least three times. Data were normally distributed as confirmed by Shapiro–Wilk test. The data are presented as the means ± SDs (n ≥ 3 biological replicates). Significant differences between groups were assessed by one-way ANOVA followed by Tukey’s HSD post hoc test. A *p* value < 0.05 was considered statistically significant.

## 3. Results

### 3.1. Differences in Fruit Quality of Mature Tomato Fruits Among Different Growth Stages

**Figure 1:**
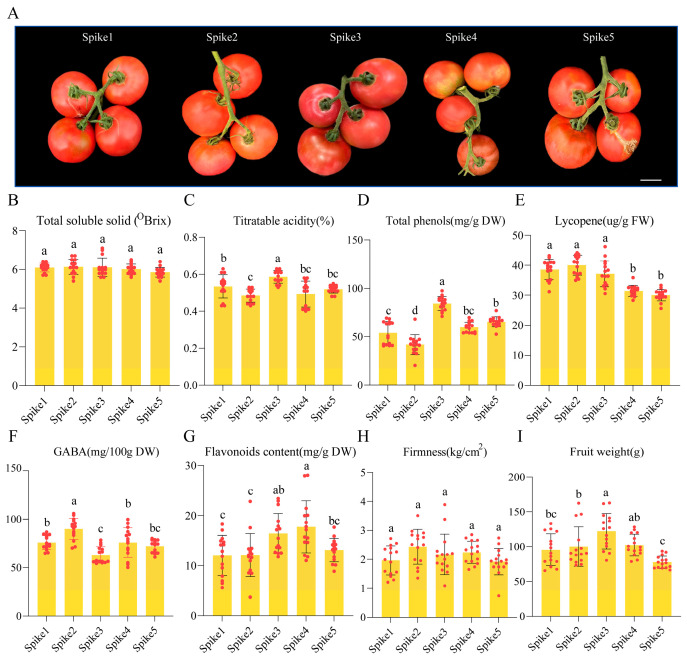
Mature tomato fruits of the 1st to 5th spikes were selected for analysis. (**A**) Mature tomato fruits from Spike1 to Spike5. Bar, 2 cm. (**B**) Total soluble solids content. (**C**) Titratable acidity. (**D**) Total phenols content. (**E**) Lycopene content. (**F**) γ-aminobutyric acid (GABA) content. (**G**) Flavonoid content. (**H**) Firmness. (**I**) Fruit weight. Fresh Weight (FW), Dry Weight (DW). A total of 15 fruits were used for each measurement, and the values shown are means ± SDs. Different lowercase letters above the bar indicate a significant difference (*p* value < 0.05, Tukey’s HSD test).

In this study, we aimed to examine the variation in fruit quality of cultivated tomatoes at the mature stage across different growth stages, each corresponding to a distinct fruit spike during extended tomato cultivation. We collected pulp tissues from Spike1 to Spike5, representing the 1st to 5th growth stages of tomato plants, respectively, at the mature stage (Figure 1A). The findings indicated that mature tomato fruits from Spike1 to Spike5 exhibited comparable levels of soluble solid content and fruit firmness (Figure 1B,H). However, significant differences were observed in titratable acid, total phenol content, gamma-aminobutyric acid (GABA) content, flavonoid content, and fruit weight of mature tomato fruits from Spike1 to Spike3 (Figure 1C,D,F–I). Notably, GABA content was highest in Spike2 (Figure 1F), while titratable acid and total phenol contents peaked in Spike3 (Figure 1C,D). Lycopene content was significantly decreased in Spike4 and Spike5 (Figure 1E). Given that the differences in the aforementioned quality indicators were more pronounced between Spike1 and Spike3 compared to Spike4 and Spike5, we conducted metabolomics and transcriptomics analyses on the mature tomatoes.

### 3.2. Metabolomes and Transcriptomes Analysis for Tomatoes from Different Growth Stages

**Figure 2:**
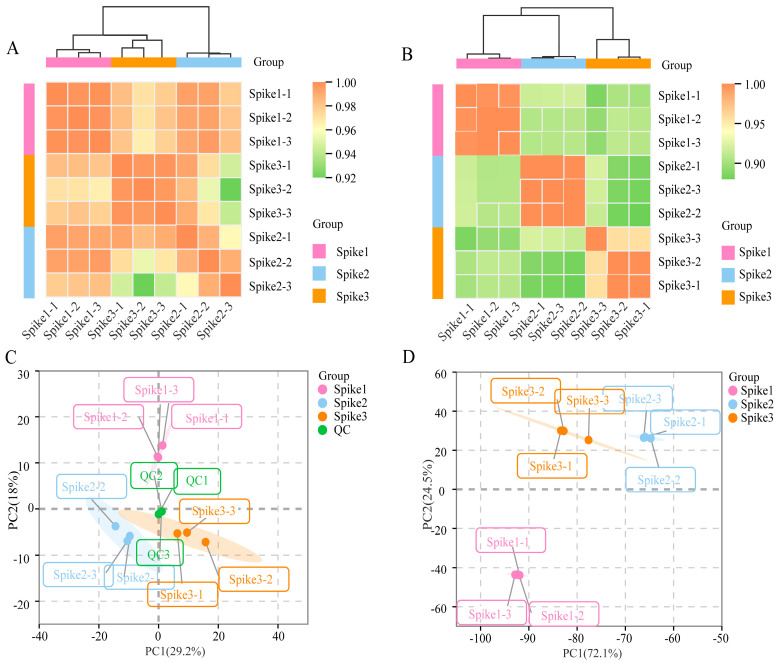
Analysis of metabolome and transcriptome data of fruit quality-related samples in mature tomato fruits from Spike1 to Spike3. (**A**) Cluster tree heat map of metabolome data. (**B**) Cluster tree heat map of transcriptome data. (**C**) Principal component analysis (PCA) of metabolome data. (**D**) PCA of transcriptome data.

A total of 872 metabolites were identified across the metabolomes of all samples (Table S1). Hierarchical clustering heatmap analysis demonstrated a clear separation among the three spike sample groups, with biological replicates within each group exhibiting tight clustering (Figure 2A,B). This finding indicates significant differences in metabolic accumulation among the three spike fruit samples, thereby reinforcing the reliability of the metabolomics data obtained. Following the removal of low-quality data from the transcriptomes, as well as ambiguous reads and adapter sequences, an average of 6.20 Gb of clean data was generated (Table S2). Additionally, a total of 248 novel unannotated genes were de novo identified from the transcriptomic data and subsequently functionally annotated after rigorous screening, thereby enhancing our understanding of the genomic framework associated with cultivated tomatoes (Table S3). In the metabolomic analysis, the expression profiles of metabolites exhibited significant differences among the three spike fruit samples, particularly between Spike1 and both Spike2 and Spike3 (Figure 2C). Notably, principal components PC1 and PC2 accounted for 96.6% of the total variance observed in the dataset (Figure 2D).

### 3.3. Integrated Transcriptomic and Metabolomic Analysis

**Figure 3:**
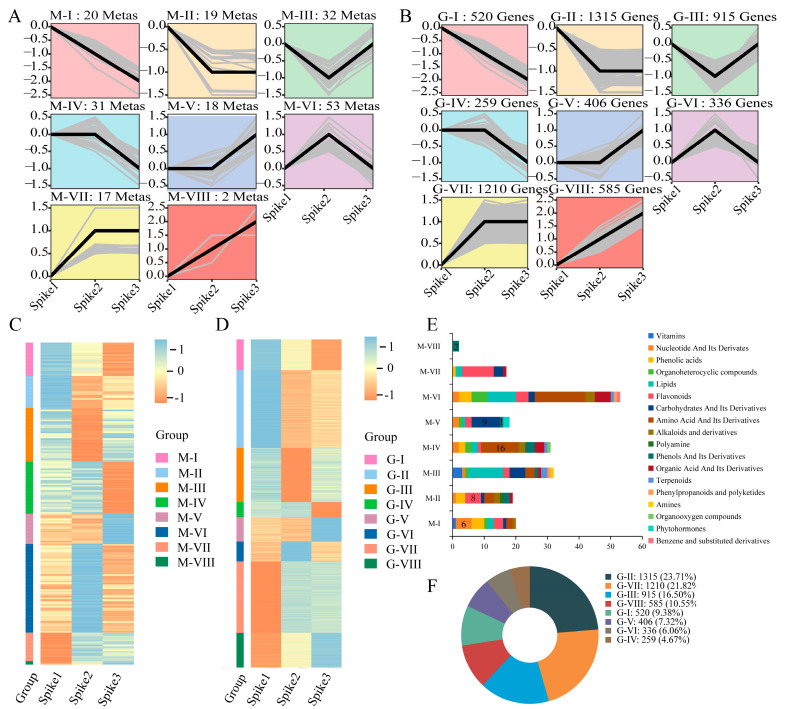
Top enrichment terms in the KEGG pathway analysis of differentially expressed genes (DEGs) and differentially accumulated metabolites (DAMs). (**A**,**B**) Dynamic pattern changes in eight groups of quality-related genes and metabolites. The *X*-axis represents mature fruits from Spike1 to Spike3, and the *Y*-axis represents the Z-scores for metabolites. The numbers represent the number of metabolites (top) and genes (top) in each group. (**C**,**D**) Metabolite modules (MMs) and Gene modules (GMs). The accumulation pattern of metabolites in eight MMs (MM-I to MM-VIII) and the expression pattern in eight GMs (GM-I to GM-VIII) are shown. The Z-scores for the dataset range from −1 to 1. (**E**) Statistics of the class categories of metabolites in eight MMs. (**F**) Percentage statistics of eight GMs.

To gain a comprehensive understanding of the metabolic profile of mature tomato fruits across different growth stages (Spike1 to Spike3), a total of 192 differentially accumulated metabolites (DAMs) were analyzed using spatiotemporal clustering (STC). The analysis identified eight distinct metabolite modules (MMs), which encompassed 18 different types of metabolites (Figure 3A,C, Table S4). These modules exhibited unique stage-specific accumulation patterns that were closely linked to fruit quality phenotypes. Specifically, within the MM-I and MM-II modules, six nucleotides and their derivatives, along with eight flavonoid metabolites, predominantly accumulated in fruits at the Spike1 (Figure 3E, Table S4). The MM-IV module was characterized by the accumulation of 16 amino acids and their derivatives in fruits at the Spike2 (Figure 3E, Table S4). Furthermore, the MM-V and MM-VIII modules were marked by the accumulation of nine carbohydrates and their derivatives, as well as three phenols and their derivatives, primarily in fruits at the Spike3 (Figure 3E, Table S4).

To enhance the comprehension of metabolic regulation alterations in mature tomato fruits from Spike1 to Spike3, a comprehensive trend analysis was conducted on 5546 differentially expressed genes (DEGs), which were subsequently categorized into eight distinct gene modules (GMs) (Figure 3B,D, Table S5). Notably, the G-II group comprises 1315 genes, representing the largest proportion at 23.71% (Figure 3B,F).

### 3.4. KEGG Enrichment Analysis

**Figure 4:**
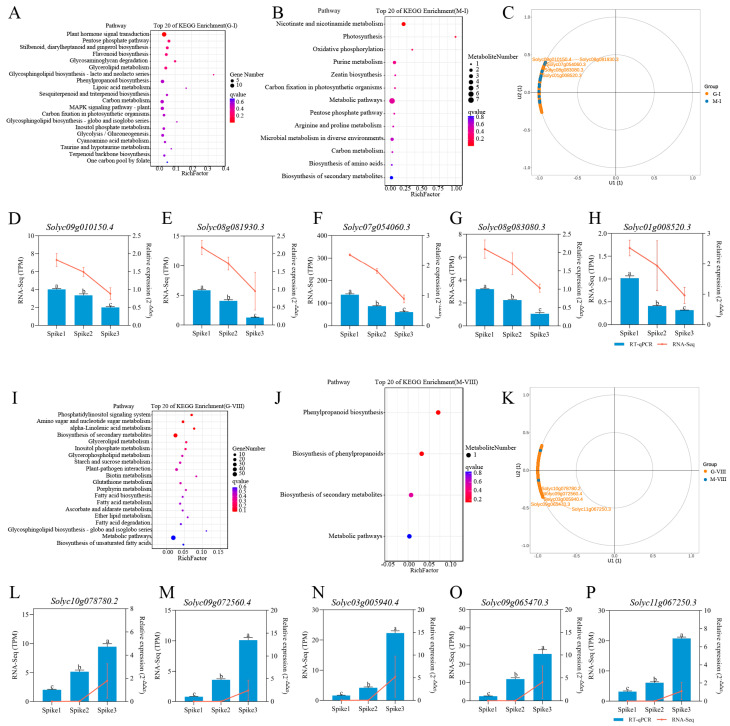
KEGG pathway enrichment in G-I, M-I, G-VIII and M-VIII. (**A**) KEGG pathway enrichment in G-I. (**B**) KEGG pathway enrichment in M-I. (**C**) The correlation between G-I and M-I. (**D**–**H**) Analysis of *Solyc09g010150.4*, *Solyc08g081930.3*, *Solyc07g054060.3*, *Solyc08g083080.3*, and *Solyc01g008520.3* expression in Spike1 to Spike3. (**I**) KEGG pathway enrichment in M-VIII. (**J**) KEGG pathway enrichment in G-VIII. (**K**) The correlation between G-VIII and M-VIII. (**L**–**P**) Analysis of *Solyc10g078780.2*, *Solyc09g072560.4*, *Solyc03g005940.4*, *Solyc09g065470.3*, and *Solyc11g067250.3* expression in Spike1 to Spike3.

A KEGG analysis was performed utilizing the eight GMs to elucidate the biological pathways that mediate the dynamics of transcripts and metabolites. The flavonoid biosynthesis pathway, identified as enriched in G-I, demonstrated a strong concordance with the accumulation of flavonoids and associated secondary metabolites in MM-I (Figure 4A,B). A correlation analysis between G-I and M-I indicated a positive relationship. Notably, the genes *Solyc09g010150.4*, *Solyc08g081930.3*, *Solyc07g054060.3*, *Solyc08g083080.3*, and *Solyc01g008520.3* exhibited the highest correlation with all metabolites in M-I (Figure 4C, Table S4). Subsequently, we analyzed the five differentially expressed genes through RT-qPCR, and found that the results were consistent with those of the transcriptome data analysis, further verifying the accuracy of the transcriptome data (Figure 4D–H). These genes are likely pivotal in mediating the observed differences in fruit substance accumulation from Spike1 to Spike3 in tomatoes. Consequently, these five genes are proposed as key regulators of the distinctive metabolite accumulation observed in Spike1 fruits. The primary enriched metabolic pathways identified in G-VIII encompassed both general metabolic pathways and the biosynthesis of secondary metabolites. These were found to be associated with the accumulation of similar pathways and metabolites in MM-VIII (Figure 4I,J). Through the calculation of the correlation between G-VIII and M-VIII, it was determined that a positive correlation exists between these two variables. Furthermore, the genes *Solyc10g078780.2*, *Solyc09g072560.4*, *Solyc03g005940.4*, *Solyc09g065470.3*, and *Solyc11g067250.3* exhibit the strongest correlation with the all metabolites present in M-VIII (Figure 4K, Table S4). Further investigation through RT-qPCR analysis of these five differentially expressed genes confirmed consistency with the transcriptome data (Figure 4L–P). These genes contribute to the progressive increase in functional metabolites observed from Spike1 to Spike3. The findings suggest that the selected DEGs and DAMs exhibit coordinated expression and accumulation patterns within their respective modules, indicating a transcript–metabolite coupling during fruit development.

### 3.5. Correlation Analysis

**Figure 5:**
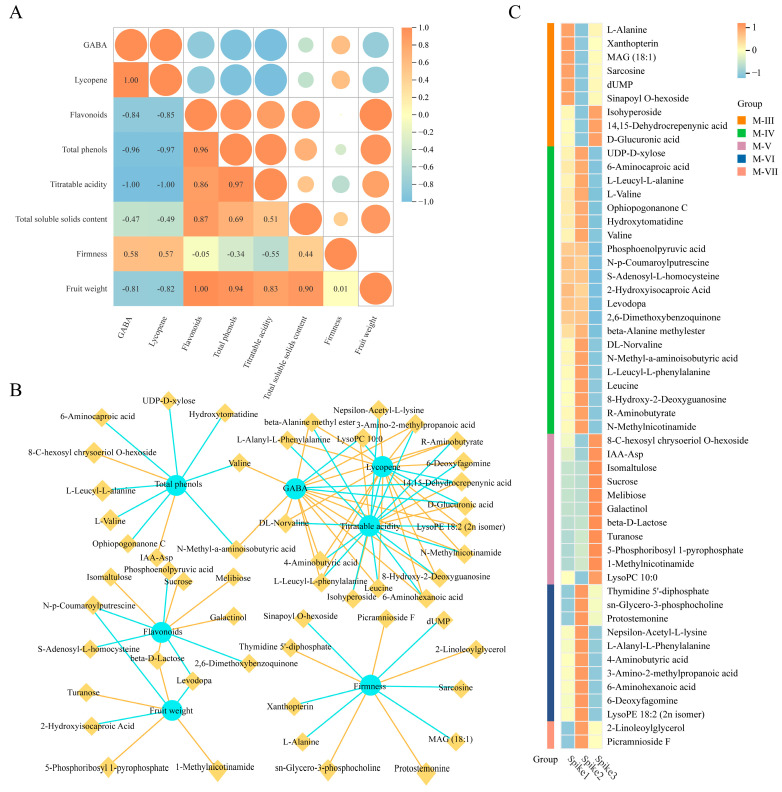
Correlation analysis. (**A**) Correlation coefficients of total soluble solids content, titratable acidity, total phenol content, lycopene content, GABA content, flavonoid content, firmness and fruit weight. (**B**) Analysis of the correlations between eight quality traits and metabolites in eight MMs. The orange dotted line represents a positive correlation, and the blue dotted line represents a negative correlation. (**C**) Heatmap of metabolites correlated with eight quality traits. The Z-scores for the dataset range from −1 to 1.

The correlation matrix demonstrated a robust positive correlation between GABA and lycopene content (PCC = 1.00), suggesting highly synchronized alterations in these two critical quality traits (Figure 5A). Among eight MMs and quality traits, several compounds, including beta-Alanine methyl ester, R-Aminobutyrate, and others, showed a strong positive correlation with GABA and lycopene (PCC > 0.8), with their levels initially rising and then falling from Spike 1 to Spike 3 (Figure 5B,C). Conversely, LysoPC 10:0, 14,15-dehydrocrepenynic acid, D-glucuronic acid, and GABA were negatively correlated with lycopene (PCC < −0.8), with their levels first decreasing and then increasing from Spike 1 to Spike 3 (Figure 5B,C). Flavonoids were strongly positively correlated with fruit weight (PCC = 1.00), indicating coordinated regulation during fruit development (Figure 5A). Beta-D-Lactose (M-V) also showed a positive correlation with both flavonoids and fruit weight (PCC > 0.8), with its levels first remaining unchanged and then increasing from Spike 1 to Spike 3 (Figure 5B,C). Conversely, N-p-Coumaroylputrescine (M-VI) and Levodopa (M-VI) were negatively correlated with flavonoids and fruit weight (PCC < −0.8), with their levels remaining stable before decreasing from Spike 1 to Spike 3 (Figure 5B,C). Total phenols and GABA content were inversely related (Figure 5A). Correlation analysis reveals that Valine (M-IV) and N-Methyl-a-aminoisobutyric acid (M-IV) were positively correlated with total phenols (PCC > 0.8) and negatively with GABA (PCC < −0.8). Titratable acid showed a strong negative correlation with GABA and lycopene (PCC = −1.00), and strong positive correlations with flavonoids (PCC = 0.86) and total phenols (PCC = 0.97) (Figure 5A). Firmness showed no significant correlation with the other seven quality traits (|PCC| < 0.8) (Figure 5A). Total soluble solids content was uncorrelated with metabolites across all eight MMs (Figure 5B,C), wuggesting that this trait is regulated independently from the major metabolic modules identified in this study.

### 3.6. Phenylpropanoid Biosynthesis and Flavonoid Biosynthesis Analysis

**Figure 6:**
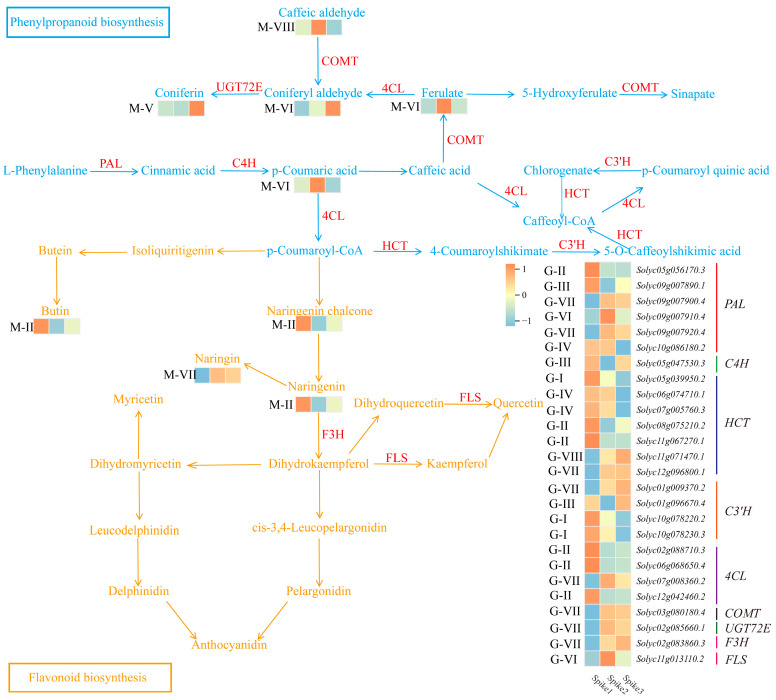
Phenylpropanoid and flavonoid biosynthesis pathways with associated metabolite and gene expression patterns. The Z-scores for the dataset range from −1 to 1. phenylalanine aminotransferase (PAL); Cinnamate-4-hydroxylase (C4H); Catechol-Omethyl transferase (COMT); 4-coumarate CoA ligase (4CL); Udp-glycosyltransferase (UGT72E); P-Coumarate 3-Hydroxylase (C3’H); Hydroxycinnamoyl-CoA shikimate/quinate hydroxycinnamoyl transferase (HCT); Flavanone 3-hydroxylase (F3H); Flavonol synthase (FLS).

In the phenylpropanoid biosynthesis process, L-phenylalanine is converted to cinnamic acid by the enzyme phenylalanine aminotransferase (PAL). Transcriptome analysis identified six *PAL* genes, with *Solyc05g056170.3* in G-II, *Solyc09g007890.1* in G-III, *Solyc09g007900.4* and *Solyc09g007920.4* in G-VII, and *Solyc09g007910.4* in G-VI. These genes exhibited four distinct expression trends from Spike1 to Spike3, which collectively determine the dynamic flux through the phenylpropanoid pathway (Figure 6). Cinnamate-4-hydroxylase (C4H) catalyzed the conversion of cinnamic acid to p-coumaric acid. The content of p-coumaric acid initially increased and then decreased from Spike1 to Spike3 consistent with the observed pattern of p-coumaric acid accumulation (Figure 6). Transcriptome analysis showed that the gene *Solyc05g530.3*, encoding C4H, initially decreased in expression and then increased from Spike1 to Spike3 (Figure 6). Catechol-Omethyl transferase (COMT) converted Caffeic acid to Ferulate, which was part of M-VI and initially increased then decreased in Spike1 to Spike3 (Figure 6). Transcriptome analysis showed the *COMT* gene, *Solyc03g080180.4*, in the G-VII group, initially increased in expression and then stabilized from Spike1 to Spike3 (Figure 6). 4-coumarate CoA ligase (4CL) catalyzed the conversion of Ferulate to Coniferyl aldehyde, which initially increased and then decreased from Spike1 to Spike3 (Figure 6). Transcriptome analysis showed that among the four 4CL genes, *Solyc07g008360.2*, followed a similar expression pattern, indicating a primary role in regulating this step (Figure 6). Udp-glycosyltransferase (UGT72E) converted Coniferyl aldehyde into Coniferin, which was part of the M-V group and initially remained constant before increasing (Figure 6). Transcriptome analysis showed that the gene *Solyc02g085660.1*, encoding UGT72E, was in the G-VII group and its expression first increased, then remained unchanged (Figure 6). P-Coumarate 3-Hydroxylase (C3’H) catalyzed the conversion of p-Coumaroyl quinic acid to Chlorogenate. Transcriptome analysis showed that among the four C3’H genes, *Solyc01g009370.2* (G-VII) increased then stabilized, *Solyc01g096670.4* (G-III) increased then decreased, and *Solyc10g078220.2* (G-I) and *Solyc10g078230.3* (G-I) continuously decreased in expression from Spike1 to Spike3. Hydroxycinnamoyl-CoA shikimate/quinate hydroxycinnamoyl transferase (HCT) facilitated the conversion of Chlorogenate to Caffeoyl-CoA (Figure 6). Transcriptome analysis identified seven *HCT* genes: *Solyc05g039950.2* (G-I), *Solyc06g074710.1* (G-IV), *Solyc07g005760.3* (G-IV), *Solyc08g075210.2* (G-II), *Solyc11g067270.1* (G-II), *Solyc11g071470.1* (G-VIII), and *Solyc12g096800.1* (G-VII). These genes exhibited five distinct expression patterns from Spike1 to Spike3, supporting complex and stage-specific regulation of the phenylpropanoid pathway (Figure 6).

In the flavonoid biosynthesis process, Butin (M-II), Naringenin chalcone (M-II), and Naringenin (M-II) initially decreased and then stabilized from Spike1 to Spike3, while Naringin (M-VII) increased before stabilizing (Figure 6). These patterns correspond directly to the stage-specific flavonoid accumulation observed in fruit quality assays. Flavanone 3-hydroxylase (F3H) catalyzed Naringenin to Dihydrokaempferol, with its gene *Solyc02g083860.3* (G-VII) showing increased expression before stabilizing (Figure 6). Flavonol synthase (FLS) converts Dihydrokaempferol to Dihydroquercetin, with its gene *Solyc11g013110.2* (G-VI) initially increasing and then decreasing in expression (Figure 6). In summary, these nine key structural gene families function coordinately to regulate phenylpropanoid and flavonoid biosynthesis, and their expression dynamics directly explain the observed changes in total phenols and flavonoids across different fruit growth stages (Spike1 to Spike3).

### 3.7. Analysis of No Significant Difference Compounds in Different Growth Stages

**Figure 7:**
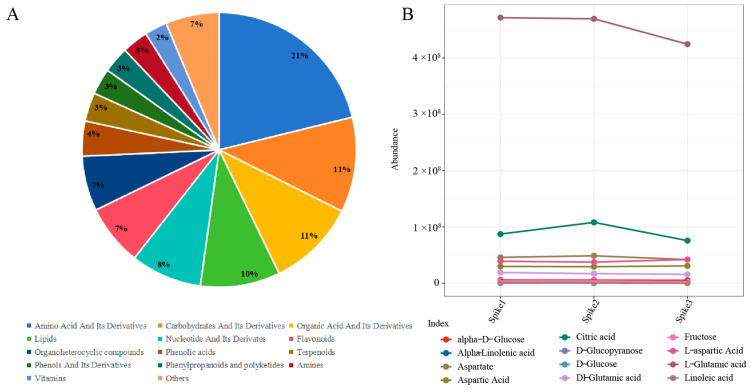
Analysis of no-significant-difference compounds in Spike1 to Spike3. (**A**) Classification of no-significant-difference compounds in Spike1 to Spike3. (**B**) The measured values of Glucose (alpha−D−Glucose, D−Glucopyranose, D−Glucose), Fructose, Citric acid, Aspartate (L−aspartic Acid, Aspartate, Aspartic Acid), Glutamic Acid (Dl−Glutamic acid, L−Glutamic acid,), Alpha−Linolenic acid, and Linoleic acid in Spike1 to Spike3.

Among the metabolic data, there were 680 metabolites that did not show any significant differences in different growth stages. These metabolites maintain relative metabolic stability during fruit growth stage. Among them, the category of amino acid and its derivatives accounted for the largest proportion, at 21% (Figure 7A). The proportions of carbohydrate and its derivative metabolites and organic acid and its derivative metabolites were 11%, respectively, while the proportion of lipid metabolites was 10% (Figure 7A). Among the amino acid and its derivative metabolites, aspartate and glutamic acid are the main metabolites that affect the flavor of tomato fruits. The contents of these two types of substances do not show significant changes in tomato Spike1 to Spike3 (Figure 7B). Among the carbohydrates and its derivatives metabolites, glucose and fructose are the main metabolites that affect the flavor of tomato fruits. The contents of these two types of substances do not show significant changes in tomato Spike 1 to Spike 3 (Figure 7B). Among the organic acid and its derivatives metabolites, the content of citric acid did not show any significant change in tomato Spike 1 to Spike 3. Among the lipid metabolites, the contents of alpha-linolenic acid and linoleic acid did not show significant changes in tomato Spike1 to Spike3 (Figure 7B). The stability of these primary and flavor-related metabolites helps maintain consistent taste and quality despite the significant shifts in secondary metabolism observed across Spike1 to Spike3.

## 4. Discussion

The quality of tomatoes is determined by several factors, including visual appeal, flavor, nutritional content, and suitability for processing and storage. A decline in flavor and nutritional quality can substantially affect the sensory attributes of the fruit, reduce consumer preference, and ultimately impact its economic value [26]. Therefore, elucidating the metabolic pathways and regulatory mechanisms involved in the synthesis and accumulation of key quality compounds is crucial for molecular breeding aimed at improving tomato fruit quality. In this study, we examined the concentrations of essential components, such as soluble solids, titratable acidity, and lycopene. By conducting an integrated analysis of transcriptomic and metabolomic data from tomatoes at various growth stages (Spike1 to Spike3) during maturation, we identified eight distinct metabolic and gene modules.

Building on previous research, it is evident that tomato fruit quality is affected by more than just the type, content, and ratio of soluble sugars and organic acids. The presence of secondary metabolites, such as polyphenols, volatile organic compounds, alkaloids, and GABA, also plays a crucial role in determining the quality of tomato fruits [27,28]. The findings indicate that the accumulation of soluble solid content in tomato fruits remains remarkably stable, suggesting that this parameter is unaffected by the growth stages. (Figure 1B). In practical production settings, maintaining consistent cultivation conditions can ensure uniform sugar content in fruits, which is of significant importance for the standardized production of commercial fruits. Conversely, functional components such as titratable acid, total phenols, GABA, and flavonoids exhibit substantial variability in their accumulation across different growth stages, categorizing them as labile indicators (Figure 1C,D,F,G). The lycopene concentration remained relatively stable at approximately 40 μg/g FW in the Spike1 to Spike3 but exhibited a significant decline in Spike4 and Spike5 (Figure 1E). This reduction may be attributed to the transcriptional repression of the carotenoid biosynthetic pathway, as evidenced by the downregulation of critical genes such as phytoene synthase and lycopene β-cyclase in fruits that develop later. These observations imply that the synthesis of these labile components is modulated by the overall metabolic activities of the plants. The accumulation patterns are intricately linked to the spatial positioning of fruit spikes and their growth stages. Consequently, a comprehensive understanding of the accumulation of these components can facilitate the precise adjustment of cultivation conditions to enhance the stability of metabolite content.

Phenolic acids, phenolic compounds and their derivatives, along with flavonoids, constitute the primary nutritional constituents in tomatoes, imparting antioxidative, lipid-regulating, and anti-tumor properties to the fruit [1,29,30]. Metabolomic analyses across different growth stages (Spike1 to Spike3) revealed the presence of 76 flavonoid compounds, 72 phenolic acids, and associated derivatives (Table S1). Among them, we selected four flavonoid compounds and five phenolic acids as the metabolites that were differentially accumulated in the fruits to characterize the accumulation trend of metabolites in the Spike1 to Spike3 tomato fruits (Figure 6). This focus on bioactive compounds aligns with broader trends in food science, where phenolic and flavonoid contents are widely recognized as key quality criteria for fruits such as pomegranate (*Punica granatum*) and blue honeysuckle (*Lonicera caerulea*) [24,31], highlighting the applicability of our quality assessment framework across different fruit species.

In the biosynthesis of phenylpropanoids, L-phenylalanine serves as the primary substrate [32]. The enzyme C4H facilitates the conversion of cinnamic acid to p-coumaric acid [33]. Notably, the expression of the gene *Solyc05g047530.3*, which encodes C4H, demonstrated an inverse relationship with the accumulation of p-coumaric acid (Figure 6). This negative correlation may indicate a metabolic feedback regulation or a post-transcriptional control mechanism, rather than a direct negative regulation of its synthesis. COMT catalyzes the transformation of caffeic acid into ferulate [34]. The gene *Solyc07g008360.2*, which encodes 4CL, also displayed a similar accumulation trend with coniferyl aldehyde, implying that it positively regulates the synthesis of this compound (Figure 6). Lastly, UGT72E is involved in the conversion of coniferyl aldehyde into coniferin [35,36]. The gene *Solyc07g008360.2* encoding 4CL had a similar accumulation trend with Coniferyl aldehyde, suggesting that *Solyc07g008360.2* positively regulates the synthesis of Coniferyl aldehyde (Figure 6). UGT72E was responsible for converting Coniferyl aldehyde into Coniferin [37]. The gene *Solyc02g085660.1* encoding UGT72E had an opposite accumulation trend with Coniferin, suggesting that *Solyc05g047530.3* negatively regulates the synthesis of Coniferin (Figure 6). In the flavonoid biosynthesis process, F3H was responsible for catalyzing the conversion of Naringenin to Dihydrokaempferol [38,39]. The gene *Solyc02g083860.3* encoding F3H had an opposite trend to the accumulation of Naringenin. It is speculated that *Solyc02g083860.3* negatively regulates the conversion of Naringenin (Figure 6). Collectively, these genotype–metabolite correlations demonstrate that differential expression of structural genes drives the distinct accumulation patterns of bioactive compounds across different growth stages (Spike1 to Spike3), providing molecular targets for quality improvement.

This study identified 680 metabolites exhibiting no significant differences in the metabolic profiles of tomato fruits from different growth stages (Spike1 to Spike3). Amino acid and its derivatives constituted the largest proportion, accounting for 21% of the metabolites (Figure 7A). These findings suggest that the core metabolic network of tomato fruits remains relatively stable throughout the ripening period [40,41]. Analysis of metabolite category distribution revealed that carbohydrates and its derivatives metabolites, organic acid and its derivatives metabolites (11%), and lipid metabolites (10%) are proportionately balanced (Figure 7A), further corroborating the conservation of the core metabolic network during tomato development. The stability of the contents of an aspartate and glutamic acid, which are sources of umami; glucose and fructose, which determine sweetness; citric, which regulates sourness; and alpha-linolenic acid and linoleic acid, which are involved in the synthesis of flavor compounds, directly suggests that the fundamental flavor profile of tomato fruits undergoes relatively minor changes during the mature stages from different growth stages (Spike1 to Spike3) (Figure 7B). This discovery aligns with the perspective that certain key flavor components in hawthorn exhibit stability throughout the ripening process [42]. Consequently, in practical production settings, it is unnecessary to intervene in the synthesis of the aforementioned related metabolite. Instead, management efforts regarding water and fertilizer, environmental control, and other practices should be redirected towards disease and pest prevention, as well as promoting nutrient accumulation in plants and other factors that influence the development of additional flavor profiles. Furthermore, these findings provide valuable insights for the improvement of tomato varieties, facilitating the targeted selection of genotypes that consistently express elevated levels of sugars, acids, or amino acids at this developmental stage, thereby enhancing the overall flavor of tomatoes.

This study is subject to several limitations inherent to its experimental design that warrant consideration. Specifically, only a single tomato cultivar (‘Sweet100’) was examined under a singular growth condition, and fruits were collected solely from Spike1 to Spike3 for omics analysis. Future research should incorporate multiple cultivars, varied growth environments, and fruits from additional fruiting trusses to assess the robustness of the identified gene–metabolite–phenotype associations.

## 5. Conclusions

Utilizing high-resolution metabolomes and transcriptomes, in combination with a joint transcriptomic and metabolomic analysis, this research developed a theoretical framework for researching the quality formation of mature tomato fruits of different growth stages (Spike1 to Spike3). The accumulation trends of these metabolites and genes are conducive to promoting quality improvements in future tomato breeding and lay a theoretical foundation for achieving long-season cultivated tomatoes with stable fruit quality.

## Data Availability

The original contributions presented in this study are included in the article/Supplementary Materials. Further inquiries can be directed to the corresponding authors.

## Supplementary Materials

The following supporting information can be downloaded at: https://www.mdpi.com/article/10.3390/foods15050883/s1, Table S1. Primary and secondary metabolites of mature “Sweet100” tomato fruits (Spike1 to Spike3); Table S2. Filtered raw transcriptome data and statistical analysis of mature “Sweet100” tomato fruits (Spike1 to Spike3); Table S3. Gene IDs prefixed with ‘MSTRG’ denote novel genes identified in this study; Table S4. Clustering results for trend analyses of primary and secondary metabolites in mature fruits (Spike1 to Spike3) of the “Sweet100” variety (MMI-MMVII); Table S5. Clustering results for differentially expressed genes from the transcriptomic analysis of mature “Sweet100” tomato fruits from Spike1 to Spike3 (GMI-GMVII).

## References

[1] Yin J., Wang K., Zhang H., Zhao Z., Li D., Liu D., Xu X., Zhao T. (2024). Analysis of the nutritional composition, biological activities, and phenolic metabolic bioactive module of cherry tomatoes. LWT.

[2] Sakthivel K., Balasubramanian R., Sampathrajan V., Veerasamy R., Appachi S.V., K K.K. (2025). Transforming tomatoes into GABA-rich functional foods through genome editing: A modern biotechnological approach. Funct. Integr. Genom..

[3] Zhu L., Zhang S., Niu Q., Li Y., Niu X., Wang P., Zhu J.-K., Lang Z. (2025). Targeted mutagenesis of SlGAD3 generates very high levels of GABA in commercial tomato cultivars. Abiotech.

[4] Takeda S., Miyasaka K., Shrestha S., Manse Y., Morikawa T., Shimoda H. (2021). Lycoperoside H, a Tomato Seed Saponin, Improves Epidermal Dehydration by Increasing Ceramide in the Stratum Corneum and Steroidal Anti-Inflammatory Effect. Molecules.

[5] Wang X., Zhang M., Wang X., Yang D., Zhang X. (2023). Synergistic regulation of the growth and fruit quality of cherry tomato by remaining fruit spikes and number of interspike leaves after pruning. Sci. Hortic..

[6] Furuta H., Qu Y., Ishizuka D., Kawabata S., Sano T., Yamori W. (2025). A novel multilayer cultivation strategy improves light utilization and fruit quality in plant factories for tomato production. Front. Hortic..

[7] Coyago-Cruz E., Corell M., Moriana A., Hernanz D., Stinco C.M., Meléndez-Martínez A.J. (2017). Effect of the fruit position on the cluster on fruit quality, carotenoids, phenolics and sugars in cherry tomatoes (*Solanum lycopersicum* L.). Food Res. Int..

[8] Li J., Di T., Bai J. (2019). Distribution of Volatile Compounds in Different Fruit Structures in Four Tomato Cultivars. Molecules.

[9] Gao Z., Khalid M., Jan F., Saeed-ur-Rahman, Jiang X., Yu X. (2019). Effects of light-regulation and intensity on the growth, physiological and biochemical properties of *Aralia elata* (miq.) seedlings. S. Afr. J. Bot..

[10] Qu Z., Qi X., Liu Y., Liu K., Li C. (2020). Interactive effect of irrigation and polymer-coated potassium chloride on tomato production in a greenhouse. Agric. Water Manag..

[11] Feng M., Zhang A., Nguyen V., Bisht A., Almqvist C., De Veylder L., Carlsbecker A., Melnyk C.W. (2024). A conserved graft formation process in Norway spruce and Arabidopsis identifies the PAT gene family as central regulators of wound healing. Nat. Plants.

[12] Shu P., Zhang Z., Wu Y., Chen Y., Li K., Deng H., Zhang J., Zhang X., Wang J., Liu Z. (2023). A comprehensive metabolic map reveals major quality regulations in red-flesh kiwifruit (*Actinidia chinensis*). New Phytol..

[13] Liu Z., Wu J., Wang L., Lu X., Ahammed G.J., Zhang X., Cui X., Wang H. (2025). Integration of transcriptome and metabolome reveals regulatory mechanisms of volatile flavor formation during tomato fruit ripening. Hortic. Plant J..

[14] Li L., Zhang H., Liu J., Huang T., Zhang X., Xie H., Guo Y., Wang Q., Zhang P., Qin P. (2023). Grain color formation and analysis of correlated genes by metabolome and transcriptome in different wheat lines at maturity. Front. Nutr..

[15] Huang X., Chu G., Wang J., Luo H., Yang Z., Sun L., Rong W., Wang M. (2023). Integrated metabolomic and transcriptomic analysis of specialized metabolites and isoflavonoid biosynthesis in *Sophora alopecuroides* L. under different degrees of drought stress. Ind. Crops Prod..

[16] Jia H., Xu Y., Deng Y., Xie Y., Gao Z., Lang Z., Niu Q. (2024). Key transcription factors regulate fruit ripening and metabolite accumulation in tomato. Plant Physiol..

[17] Nishiyama T., Sulistyaningdyah W.T., Ueda K., Kusakabe H. (2020). GABA enzymatic assay kit. Biosci. Biotechnol. Biochem..

[18] Kim J.Y., Kim D.H., Kim M.S., Jung Y.J., Kang K.K. (2024). Physicochemical Properties and Antioxidant Activity of CRISPR/Cas9-Edited Tomato SGR1 Knockout (KO) Line. Int. J. Mol. Sci..

[19] Lasunon P., Sengkhamparn N. (2022). Effect of Ultrasound-Assisted, Microwave-Assisted and Ultrasound-Microwave-Assisted Extraction on Pectin Extraction from Industrial Tomato Waste. Molecules.

[20] Miranda M., Sun X., Marín A., Dos Santos L.C., Plotto A., Bai J., Benedito Garrido Assis O., David Ferreira M., Baldwin E. (2022). Nano- and micro-sized carnauba wax emulsions-based coatings incorporated with ginger essential oil and hydroxypropyl methylcellulose on papaya: Preservation of quality and delay of post-harvest fruit decay. Food Chem. X.

[21] Tai Z., Zheng M., Yang Y., Xie C., Li Z., Xu C. (2023). Temperature controlled microcapsule loaded with Perilla essential oil and its application in preservation of peaches. Front. Nutr..

[22] Robinson M.D., McCarthy D.J., Smyth G.K. (2010). edgeR: A Bioconductor package for differential expression analysis of digital gene expression data. Bioinformatics.

[23] Love M.I., Huber W., Anders S. (2014). Moderated estimation of fold change and dispersion for RNA-seq data with DESeq2. Genome Biol..

[24] Shen Y., Wang S., Wan R., Jiao J., Zhao Y., Cong L., Zhang K., Hao P., Liu Y., Xu W. (2026). Integrated transcriptomic and physiological analysis highlights the candidate genes regulating enzymatic browning, antioxidant capacity, and lipid metabolism in pomegranate aril browning. Postharvest Biol. Technol..

[25] Kanehisa M., Sato Y., Kawashima M., Furumichi M., Tanabe M. (2016). KEGG as a reference resource for gene and protein annotation. Nucleic Acids Res..

[26] Wang D., Seymour G.B. (2017). Tomato Flavor: Lost and Found?. Mol. Plant.

[27] Alseekh S., Tohge T., Wendenberg R., Scossa F., Omranian N., Li J., Kleessen S., Giavalisco P., Pleban T., Mueller-Roeber B. (2015). Identification and mode of inheritance of quantitative trait loci for secondary metabolite abundance in tomato. Plant Cell.

[28] Lee J., Nonaka S., Takayama M., Ezura H. (2018). Utilization of a Genome-Edited Tomato (*Solanum lycopersicum*) with High Gamma Aminobutyric Acid Content in Hybrid Breeding. J. Agric. Food Chem..

[29] Romagnolo D.F., Selmin O.I. (2012). Flavonoids and cancer prevention: A review of the evidence. J. Nutr. Gerontol. Geriatr..

[30] Zhou L., Sun Z., Hu T., Chen D., Chen X., Zhang Q., Cao J., Zhu B., Fu D., Zhu H. (2024). Increasing flavonoid contents of tomato fruits through disruption of the SlSPL-CNR, a suppressor of SlMYB12 transcription activity. Plant Biotechnol. J..

[31] Chen J., Fu C., Wang H., Sun X., Ma K., Yang H., Qin D., Huo J., Gang H. (2025). Combination transcriptomic and metabolomic reveal deterioration of the blue honeysuckle (*Lonicera caerulea* L.) fruit and candidate genes regulating metabolism in the post-harvest stage. Int. J. Biol. Macromol..

[32] Geng D., Shen X., Xie Y., Yang Y., Bian R., Gao Y., Li P., Sun L., Feng H., Ma F. (2020). Regulation of phenylpropanoid biosynthesis by MdMYB88 and MdMYB124 contributes to pathogen and drought resistance in apple. Hortic. Res..

[33] Bibi G., Shafique I., Ali S., Ahmad R., Shah M.M., Naqvi T.A., Zeb I., Maathuis F.J.M., Hussain J. (2024). Cyclic guanosine monophosphate improves salt tolerance in Solanum lycopersicum. J. Plant Res..

[34] Zhen Z., Xiang L., Li S., Li H., Lei Y., Chen W., Jin J.M., Liang C., Tang S.Y. (2025). Designing a whole-cell biosensor applicable for S-adenosyl-l-methionine-dependent methyltransferases. Biosens. Bioelectron..

[35] Ehlting J., Büttner D., Wang Q., Douglas C.J., Somssich I.E., Kombrink E. (1999). Three 4-coumarate:coenzyme A ligases in Arabidopsis thaliana represent two evolutionarily divergent classes in angiosperms. Plant J. Cell Mol. Biol..

[36] Lin C.-Y., Sun Y., Song J., Chen H.-C., Shi R., Yang C., Liu J., Tunlaya-Anukit S., Liu B., Loziuk P.L. (2021). Enzyme Complexes of Ptr4CL and PtrHCT Modulate Co-enzyme A Ligation of Hydroxycinnamic Acids for Monolignol Biosynthesis in *Populus trichocarpa*. Front. Plant Sci..

[37] Wu J., Zhu W., Shan X., Liu J., Zhao L., Zhao Q. (2022). Glycoside-specific metabolomics combined with precursor isotopic labeling for characterizing plant glycosyltransferases. Mol. Plant.

[38] Tu Y., Liu F., Guo D., Fan L., Zhu Z., Xue Y., Gao Y., Guo M. (2016). Molecular characterization of flavanone 3-hydroxylase gene and flavonoid accumulation in two chemotyped safflower lines in response to methyl jasmonate stimulation. BMC Plant Biol..

[39] Singh K., Rani A., Kumar S., Sood P., Mahajan M., Yadav S.K., Singh B., Ahuja P.S. (2008). An early gene of the flavonoid pathway, flavanone 3-hydroxylase, exhibits a positive relationship with the concentration of catechins in tea (*Camellia sinensis*). Tree Physiol..

[40] Klie S., Osorio S., Tohge T., Drincovich M.F., Fait A., Giovannoni J.J., Fernie A.R., Nikoloski Z. (2014). Conserved changes in the dynamics of metabolic processes during fruit development and ripening across species. Plant Physiol..

[41] Biais B., Bénard C., Beauvoit B., Colombié S., Prodhomme D., Ménard G., Bernillon S., Gehl B., Gautier H., Ballias P. (2014). Remarkable reproducibility of enzyme activity profiles in tomato fruits grown under contrasting environments provides a roadmap for studies of fruit metabolism. Plant Physiol..

[42] Wang Y., Hao R., Guo R., Nong H., Qin Y., Dong N. (2023). Integrative Analysis of Metabolome and Transcriptome Reveals Molecular Insight into Metabolomic Variations during Hawthorn Fruit Development. Metabolites.

